# Reversible reddish skin color change in a patient with compressive radial neuropathy

**DOI:** 10.1186/s12883-018-1128-y

**Published:** 2018-08-21

**Authors:** Jong Hyeon Ahn, Dae Joong Kim, Jung-Joon Sung, Yoon-Ho Hong, Suk-Won Ahn, Jeong Jin Park, Byung-Nam Yoon

**Affiliations:** 1 Department of Neurology, Inha University Hospital, Inha University College of Medicine, Incheon, South Korea; 2 Department of Anatomy, Inha University College of Medicine, Incheon, South Korea; 3 Department of Neurology, Seoul National University Hospital, Seoul, South Korea; 4 Department of Neurology, Seoul National University College of Medicine, Seoul National University Seoul Metropolitan Government Boramae Medical Center, Seoul, South Korea; 5 Department of Neurology, Chung-Ang University Hospital, Chung-Ang University College of Medicine, Seoul, South Korea; 6 Department of Neurology, Konkuk University Medical Center, Seoul, South Korea; 7 Department of Neurology, Seoul Paik Hospital, Inje University College of Medicine, Mareunnae-ro 9, Jung-gu, Seoul, 04551 Republic of Korea

**Keywords:** Radial neuropathy, Nerve compression syndrome, Autonomic dysreflexia, Sympathetic nervous system, Postganglionic sympathetic fibers, Red skin pigment

## Abstract

**Background:**

The motor and sensory symptoms caused by compressive radial neuropathy are well-known, but the involvement of the autonomic nervous system or the dermatologic symptoms are less well known. We report an unusual case of compressive radial neuropathy with reversible reddish skin color change.

**Case presentation:**

A 42-year-old man was referred for left wrist drop, finger drop and a tingling sensation over the lateral dorsum of the left hand. Based on clinical information, neurologic examinations and electrophysiologic studies, he was diagnosed with compressive radial neuropathy. In addition, a reddish skin color change was observed at the area of radial sensory distribution. After two weeks of observation without specific treatment, the skin color had recovered along with a marked improvement in weakness and aberrant sensation.

**Conclusions:**

Compressive radial neuropathy with a reversible reddish skin color change is unusual and is considered to be due to vasomotor dysfunction of the radial autonomic nerve. Compressive radial neuropathy is presented with not only motor and sensory symptoms but also autonomic symptoms; therefore, careful examination and inspection are needed at diagnosis.

## Background

For compressive neuropathy in the upper extremities, radial neuropathy is as frequent as median and ulnar neuropathy [1]. Compressive radial neuropathy commonly occurs at the spiral groove and results in various symptoms, such as marked wrist drop and finger drop due to denervation of extensor muscles and mild weakness of the supinator muscle. Sensory disturbance is present in the distribution of the superficial radial sensory nerve (SRN). The motor and sensory symptoms caused by compressive radial neuropathy are well known, but the involvement of the autonomic nervous system or the dermatologic symptoms are less well known. Diverse dermatologic symptoms have been reported in carpal tunnel syndrome (CTS), including ulceration, blistering, hypohidrosis, Raynaud’s phenomenon, and irritant contact dermatitis [2], but these symptoms are rare in compressive radial neuropathy. Here, we report an unusual case of compressive radial neuropathy with reversible reddish skin color change.

## Case presentation

**Fig. 1:**
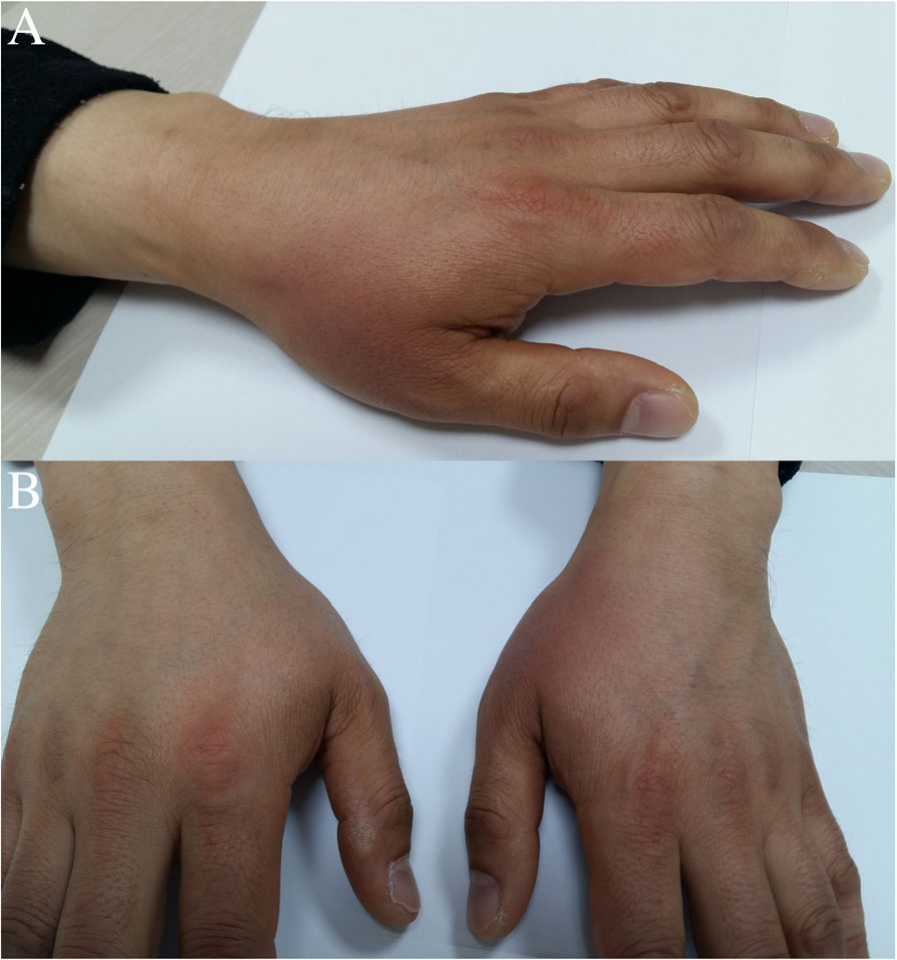
Hands 4 days after onset. **a** Reddish skin observed at the lateral dorsum of the left hand between the thumb and index finger extending into the proximal phalanges of the 2nd finger dorsum. **b** Left hand shows a definite color change compared to the right hand

A 42-year-old male was referred for left wrist drop, finger drop and a tingling sensation over the lateral dorsum of the left hand. The patient reported that he was well until 4 days prior when he was intoxicated and awoke with the symptoms. For 4 days, slight improvement of weakness occurred. He had no history of antecedent trauma, injury, infection, or mononeuropathy. Neurologic examination revealed weakness of the left wrist and finger extension (Medical Research Council grade II). Finger abduction appeared weak, but strength improved when the hand was passively extended to the neutral position. Wrist and finger flexion was intact. On sensory examination, there was a well-demarcated area of hypoesthesia and a tingling sensation over the lateral dorsum of the left hand between the thumb and index finger extending into the proximal phalanges of the 2nd finger. In addition, reddish skin color and slight edema were observed in the same area (Fig. 1). There was no definite change in skin temperature and no pain. Reflexes were normal at the biceps and triceps brachii muscles, but the left brachioradialis reflex was absent. Routine blood analysis showed white blood cell count, C-reactive protein level and uric acid level were normal. According to the clinical information and neurologic examination, he was diagnosed with compressive radial neuropathy. After approximately two weeks of observation without specific treatment, the skin color recovered along with a marked improvement of the weakness and aberrant sensation. A nerve conduction study and electromyography were performed 2 weeks after the onset of the symptoms (Table 1). On the affected left side, a normal radial compound motor action potential (CMAP) was recorded over the extensor indicis proprius muscle with the forearm and elbow stimulated. When stimulated above the spiral groove, the CMAP was reduced by 34% compared to that of distal stimulations. The contralateral radial motor nerve study and sensory nerve conduction were normal. Electromyography revealed that the left extensor indicis proprius, extensor digitorum communis, extensor carpi radialis and brachioradialis showed increased insertional activity, fibrillation potentials, and positive sharp waves and reduced recruitment pattern.

**Table 1:** The results of nerve conduction study and electromyography at 2 weeks after the onset

Nerve	Stimulation	Latency (msec)	Amp.	Velocity (m/sec)	F-latency (msec)	
Motor						
Lt. radial	Forearm	1.75 (< 2.0)	5.9			
	Elbow		5.8	58.7 (> 49.0)		
Above	spiral groove		3.9*	53.5 (> 49.0)		
Rt. radial	Forearm	1.64 (< 3.6)	6.6			
	Elbow		6.4	57.9 (> 49.0)		
Above	spiral groove		6.4	58.1 (> 49.0)		
Lt. median	Wrist	2.88 (< 3.6)	11.9 (> 5.0)		26.9	
	Elbow		12.3	61.3 (> 50.0)		
	Axilla		12.0	67.9 (> 56.0)		
Rt. median	Wrist	3.33 (< 3.6)	10.9 (> 5.0)		27.7	
	Elbow		10.3	62.5 (> 50.0)		
	Axilla		9.8	61.3 (> 50.0)		
Sensory						
Lt. radial	Forearm	4.27	10.8 (> 8.0)	53.9 (> 50.0)		
Rt. radial	Forearm	4.19	9.4 (> 8.0)	54.9 (> 50.0)		
Lt. median	Digit 2	2.48	27.2 (> 10.0)	44.4 (> 41.3)		
Rt. median	Digit 2	2.54	21.3 (> 10.0)	43.3 (> 41.3)		
Electromyography	Spontaneous activity			Voluntary contraction		
Muscle (Left)	Insertional Activity	Fibrillation potentials	Positive sharp waves	Activation	Recruitment pattern	MUP morphology
EIP	↑	+ 1	+ 1	NL	↓	NL
EDC	↑	+ 2	+ 2	NL	↓	NL
Extensor carpi radialis	↑	+ 1	+ 2	NL	↓	NL
Brachioradialis	↑	+ 1	+ 1	NL	↓	NL
Triceps brahii	NL	0	0	NL	NL	NL
Deltoid	NL	0	0	NL	NL	NL
Biceps brachii	NL	0	0	NL	NL	NL
Abductor pollicis brevis	NL	0	0	NL	NL	NL
First dorsal interosseous	NL	0	0	NL	NL	NL
Cervical paraspinal C5-C7	NL	0	0	NL	NL	NL

* reduced 34% compared to that of distal stimulation. Latencies are in milliseconds, amplitudes of compound muscle action potentials in millivolts; amplitudes of sensory nerve action potentials in microvolts; velocities in meters/sec; Amp = amplitude; Lt = left; Rt = right; MC = musculocutaneous; EIP = extensor indicis propius; EDC = extensor digitorum communis;↑ = Increased; ↓ = reduced; NL = normal

## Discussion and conclusion

Most peripheral nerves are mixed nerves consisting of motor, sensory and autonomic nerve fibers. The radial nerve begins as the terminal branch of the posterior cord of the brachial plexus. The radial nerve then travels distally and bifurcates into the posterior interosseous nerve (PIN) and SRN branches. Compression of the PIN presents pure motor symptoms, such as wrist and finger drop, with variable weakness of wrist extension and radial deviation of the extended wrist. SRN compression results in pain or dysesthesias on the dorsal radial forearm radiating to the thumb and index finger. Motor weakness and sensory symptoms usually occur together if the compression site is located before the bifurcation. In this case, the patient presented with a reddish skin color change, which suggested autonomic nervous system abnormality caused by vasodilation. These kinds of autonomic dysfunctions are reported in CTS but rarely reported in radial neuropathy [3]. One study reported that 54.7% of idiopathic CTS cases had autonomic dysfunction. Of the 76 cases, 59% had painful swelling of the fingers, 39% dry palms, 33% Raynaud’s phenomenon, and 32% blanching of fingers. A reddish skin color change is also observed in patients with acute phase complex regional pain syndrome (CRPS). In CRPS, inhibition of sympathetic vasoconstrictor activity leads to vasodilation and skin warming [4]. In our case, similar to CRPS, sympathetic dysfunction caused by radial nerve compression may induce vasodilation and reddish skin innervated by the radial nerve. As the radial motor and sensory symptoms improve, the skin color improves. With a reddish skin color change, an elevated skin temperature could be suspected but not evident. Here, the skin thermometer test was not performed. In one cadaver study, the distribution of the sympathetic fibers of the radial nerve in the forearm was reported [5]. Studies on autonomic nerves of the radial nerve are still lacking.

Autonomic symptoms in radial neuropathy are unusual. Vasomotor innervation of skin of the radial dorsum of the hand is normally provided by the median nerve, whereas sensory and sudomotor innervation is received via the radial nerve [6]. In a study using local anesthetic nerve block, only one of 18 subjects had vasomotor innervation via the radial nerve.

To the best of our knowledge, this is the first report of compressive radial neuropathy with a reversible reddish skin color change. Compressive radial neuropathy is presented with not only motor and sensory symptoms but also autonomic symptoms; therefore, careful examination and inspection are needed at diagnosis.

## Abbreviations

CMAP: compound motor action potential
CRPS: complex regional pain syndrome
CTS: carpal tunnel syndrome
PIN: posterior interosseous nerve
SRN: superficial radial sensory nerve
